# Prospective Predictors of Adolescent Screen Time and Problematic Screen Use

**DOI:** 10.1016/j.jaacop.2025.06.007

**Published:** 2025-07-03

**Authors:** Beatrice A. Grund, Monica Luciana

**Affiliations:** a University of Minnesota, Minneapolis, Minnesota

**Keywords:** addiction, digital media, impulsivity, motivation, psychopathology

## Abstract

**Objective:**

Adolescent digital media use is highly prevalent. However, potential harms are unclear, as prospective studies of outcomes of screen-naïve youth are sparse. This study assessed whether individual differences in 4 domains relevant to addiction, measured in late childhood, prospectively predicted average daily hours of screen time (ST) and problematic screen use (PSU), defined as screen use that is compulsive and distressing, of 3 screen activities in mid-adolescence.

**Method:**

Youth (N = 4,754) with available data at the baseline and year 4 waves of the Adolescent Brain Cognitive Development℠ (ABCD) Study (data release 5.1) were included. Independent linear mixed-effects regression models tested whether facets of impulsivity, motivation, psychopathology, and cognitive ability at ages 9 to 10, when screen use was minimal, predicted ST and PSU of video games, social media, and smartphones at ages 12 to 15, controlling for demographics and nesting participants within families and study sites.

**Results:**

ST in mid-adolescence was positively predicted by baseline urgency, a facet of impulsivity, as well as reward sensitivity and externalizing. ST was negatively predicted by cognitive ability. Across all measured screen activities, PSU at ages 12 to 15 was positively predicted by urgency and punishment sensitivity, but not by cognitive abilities. PSU of video games and smartphones was predicted by externalizing. In mid-adolescence, ST and PSU of video games was predicted by internalizing.

**Conclusion:**

Engaging with digital media for many hours each day does not necessarily imply problematic use. Problematic screen use in mid-adolescence appears uniquely predicted by traits related to anxious distress. Comparisons with other addictive behaviors are discussed.

**Clinical guidance:**

• Routinely assess the healthiness of young patients’ screen use behaviors in mental health contexts.

• Initiate open-ended conversations with child and adolescent patients about their feelings about screen media.

• Encourage parents of adolescents to consider monitoring the screen use of their children, especially children with pre-existing mental health challenges.

## Plain language summary

This study used personality, cognitive performance, mental health, and screen use data from thousands of young American adolescents tracked in their early teens as part of the Adolescent Brain Cognitive Development℠ (ABCD) Study. The study found that 9- and 10-year-olds who report greater tendencies towards impulsivity when they feel strong emotions tend to report more problematic use of screen media (video games, social media, and mobile phones) four years later. Some traits that are known to increase people’s risk for drug addictions were related to problematic screen use, but others were not. This study suggests that young people who use screen media for a long time might not necessarily be “addicted” to their screens; rather, some young screen users who are already at risk for addictive behaviors might be more likely to develop a problem with screens.

American youth spend considerable time on digital media. Currently, American teenagers report spending an average of 8½ hours each day on recreational screen time, including time spent on video games, social media, smartphones, and other platforms.^1^ Whether increased average daily screen time (ST) hours are harmful is debated. Whereas some meta-analyses have found that youth with greater ST hours show slightly greater internalizing and externalizing problems,^2^ others suggest that impacts are minimal.^3^ Causal interpretations are frequently offered, but may be erroneous because prospective studies of screen-naïve youth are sparse. Accordingly, whether engagement with digital media for long hours each day directly leads to harmful outcomes is unknown. It is also unknown whether potential harms of prolonged ST vary by screen activity, content of screen use, motivation for screen use, or screen user characteristics, such as premorbid psychopathology.

Heavy screen use is often described as an addiction.^4^ For some digital media users, regardless of quantity of ST, screen use is compulsive, distressing, and impairing, qualifying as problematic screen use (PSU)^5^ and resembling forms of addiction such as substance use disorders (SUDs).^6^ Similar to the term SUDs, which encompasses problematic use of alcohol, cannabis, and other drugs,^6^ the term PSU encompasses problematic use of various screen-based activities involving internet usage. In contrast to ST, which is simply the total average hours of screen use across days of the week, PSU is characterized by intentional mood modification, high salience (ie, screen use is highly compelling), tolerance (gradual habituation to rewarding screen stimuli), withdrawal (agitation during screen unavailability), conflict (interpersonal or functional consequences of screen use), and relapse (resuming screen use after attempted abstinence).^7^ Characteristics of PSU shared with SUDs include preoccupation, withdrawal, and other symptoms.^8^ Additionally, recent longitudinal work found associations between time spent engaging in digital media activities and substance experimentation in early adolescence.^9^ Such evidence raises questions of whether PSU and SUDs share etiological features,^10^ including premorbid risk factors such as impulsivity, reward or punishment sensitivity, externalizing, and lower cognitive abilities.^11^

These risk factors may be expressed differently for different forms of PSU. Video game PSU has been associated with massive time displacement and suicidal ideation^12^; social media PSU has been associated with fear of missing out and anxiety over disconnection^13^; and smartphone PSU has been associated with anxiety, disrupted sleep, and depression.^14^ Despite obvious strengths, such as the use of large diverse samples, many PSU studies are cross-sectional and correlational, limiting the extent to which causal inferences can be drawn, but perhaps drawing attention to areas that merit further investigation.^15^, ^16^, ^17^ Yet conducting randomized controlled trials assessing harms of screen use is highly impractical. Thus, establishing causal associations is challenging. Moreover, most youth using screens—even for lengthy periods—may do so without problems,^18^ with harms limited to a subset of vulnerable users. Addiction research is plagued by questions about whether adverse outcomes evident in the context of active use represent antecedent vulnerabilities or consequences of engagement with the addictive substance or behavior.^19^ This is a critical issue that must be resolved in relation to ST and PSU if screen use is to be conceptualized as a salient public health concern. It is unclear whether increased screen use leads to adverse behavioral outcomes or whether individuals with certain characteristics are more likely to engage heavily with screens, leading to greater risk for PSU.

This article contributes to the current literature by improving understanding of risk factors of PSU, which may inform prevention and intervention efforts. This analysis focuses on 4 behavioral domains that are associated with the development of SUDs and may therefore be relevant to PSU, given that distinct forms of addiction are hypothesized to have shared etiologies.^20^

Impulsivity, the tendency to act rashly despite negative long-term consequences, strongly predicts SUD development^21^ and may predict PSU. Cross-sectional studies find more impulsive adolescents to have higher rates of problematic video game use,^15^ higher problematic social media use scores,^16^ higher rates of dependence on mobile phones,^22^ and higher proneness to smartphone addiction^17^ than peers. These associations could be explained by the difficulty that impulsive youth experience in regulating behaviors with negative consequences. However, existing findings do not indicate which aspects of impulsivity are most linked to PSU. Impulsivity is expressed through several distinct yet interrelated subfacets: urgency (tendencies toward rash action in response to strong affect), sensation-seeking (tendencies toward thrill-seeking), lack of planning (acting without considering consequences), and lack of perseverance (low sustained attention abilities).^23^ Examining subfacets of impulsivity could highlight similarities between PSU and SUDs. Specifically, high urgency is a marker of comorbid psychopathology, whereas sensation-seeking distinguishes SUDs from other forms of psychopathology.^24^ If PSU is strongly predicted by urgency, this would suggest emotional coping to be central to its development. If PSU is predicted by lack of perseverance or sensation-seeking, these associations would implicate arousal- or novelty-seeking as possible motivations.

Other potentially relevant SUD risk factors include motivational dispositions, such as reward sensitivity (RS), the tendency to approach appetitive stimuli, and punishment sensitivity (PS), the tendency to avoid aversive stimuli.^25^^,^^26^ Higher RS is associated with more frequent adolescent substance use,^25^ and PS is associated with increased odds of SUD in young adults.^27^ Youth with high RS could be more responsive to rewarding points, likes, and notifications associated with screen use, perhaps leading to longer average daily ST. Existing research on RS and screen use is mixed and suggests differential associations with different screen activities. Although heightened sensitivity to digital rewards may increase social media posting behaviors,^28^ decreased neural RS has been associated with greater problematic video game use.^29^ PS could also predict PSU: youth who avoid threats in offline contexts could use digital escapism to cope with distress. Concordantly, cross-sectional research shows that young adults with higher PS have higher scores on measures of problematic video game use.^30^

Concerning psychopathology, externalizing tendencies are strongly associated with SUD development, as are internalizing tendencies to a lesser degree.^31^ If externalizing (and not internalizing) predicts PSU, PSU treatments may be most effective when targeting disinhibition.

Finally, lower executive functioning (EF) abilities are longitudinally associated with greater adolescent substance use^32^ and could likewise be a PSU risk factor. Furthermore, examining associations between EF and screen use could further clarify associations between impulsivity and screen use.^15^, ^16^, ^17^

Across these 4 domains, it is unclear whether characteristics that predict PSU will also predict average daily ST. Characteristics that predict PSU merit further study for several reasons. First, research distinguishing ST-specific predictors from PSU-specific predictors is scarce, and spending a long time engaged in screen activities is not necessarily problematic. Assessing shared and specific risk factors for ST and PSU may inform efforts to prevent PSU and target interventions, including parental monitoring of youth at greatest risk.^33^ Second, existing measurement of screen use varies widely by study, as some studies examine only associations for 1 type of screen use (eg, social media use). Using well-validated and highly similar scales to measure PSU risk factors among different screen activities within the same sample may allow for more nuanced conclusions about activity-specific PSU risks. Third, measuring characteristics before onset of PSU can illuminate which are antecedents of PSU. Accordingly, this study addresses 2 primary research questions. First, do late childhood individual difference factors known to be related to SUDs also predict mid-adolescent PSU and/or ST? Second, do these associations vary by activity (video games, social media, phones)?

We hypothesize that PSU in mid-adolescence will be predicted by late-childhood traits related to disinhibition and reward-seeking: higher impulsivity subfacets, higher externalizing, higher RS, and lower EF scores. PSU may also be predicted by internalizing psychopathology. Because increased screen use is associated with internalizing symptoms,^2^ we hypothesize that ST will also be predicted by internalizing scores. These predictive associations may vary by screen activity; these latter analyses are considered exploratory.

## Method

### Sample

Secondary analyses were conducted on data from the Adolescent Brain Cognitive Development℠ (ABCD) Study (Release 5.1), a large, longitudinal cohort study with recruitment from 21 US sites. Detailed information on the recruitment procedures, inclusion/exclusion criteria, and assessment batteries of the ABCD® Study are provided elsewhere.^34^^,^^35^ Participants were recruited at ages 9 to 10 from 2016 to 2018 and are being followed longitudinally. Release 5.1 includes data from all 11,875 ABCD Study participants at the baseline through year 3 waves as well as 4,744 participants at year 4. These 4,744 youths comprise the sample of the current study (Table 1). Centralized institutional review board approval was received, as were parental consent and participant assent at each assessment wave.

**Table 1:** Demographic, Individual Difference, and Screen Use Summary Statistics

Measure	Value	
	**Mean**	**(SD)**
Sample demographics		
Age, y		
Baseline, range: 8-11	9.51	(0.50)
Year 4, range: 11-17	13.64	(0.73)
	**%**	
Sex assigned at birth		
Male	52.34	
Female	47.64	
Race/ethnicity		
Asian	2.31	
Black	10.71	
Hispanic	20.59	
Other	9.97	
White	56.42	
Maximum parental education		
Less than high school	2.53	
High school degree or GED	5.64	
Associate’s degree or some college	23.20	
Bachelor’s degree	29.00	
Master’s degree or higher	39.63	
Refused to answer	0.06	
Household income		
<$50,000	24.64	
$50,000-$99,999	27.34	
≥$100,000	40.67	
Refused to answer or do not know	7.36	
	**Mean**	**(SD)**
Baseline individual differences		
Urgency subscale score, possible score range: 8-32	16.20	(4.76)
Sensation-Seeking subscale score, possible score range: 4-16	9.80	(2.67)
Lack of Planning subscale score, possible score range: 4-16	7.69	(2.36)
Lack of Perseverance subscale score, possible score range: 4-16	6.97	(2.22)
BAS Reward Responsiveness + Drive score, possible score range: 0-24	13.01	(4.58)
BIS total score, possible score range: 0-12	5.52	(2.82)
CBCL Internalizing total raw score, possible score range: 0-50	4.23	(5.49)
CBCL Externalizing total raw score, possible score range: 0-50	5.01	(5.42)
General cognitive ability component score	0.07	(0.77)
EF component score	0.06	(0.75)
Learning/memory component score	0.07	(0.69)
	**Mean**	**(SD)**
Baseline and year 4 screen use		
Baseline ST		
Average daily video game ST, h	0.99	(1.07)
	**%**	
Does not play video games	12.92	
Plays video games for ≤30 min/day	49.69	
	**Mean**	**(SD)**
Average daily social media ST, h	0.10	(0.36)
	**%**	
Does not use social media	82.65	
Uses social media for ≤30 min/day	95.86	
	**Mean**	**(SD)**
Year 4 ST		
Average daily video game ST, h	2.75	(3.61)
	**%**	
Does not play video games	27.22	
Plays video games for ≤30 min/day	35.84	
	**Mean**	**(SD)**
Average daily social media ST, h	1.59	(2.20)
	**%**	
Does not use social media	38.31	
Uses social media for ≤30 min/day	45.02	
Owns a smartphone/smartwatch	89.10	
	**Mean**	**(SD)**
Year 4 PSU		
VGAQ score, range: 6-36, PSU cutoff = 24	12.82	(6.23)
SMAQ score, range: 6-36, PSU cutoff = 24	11.72	(5.56)
MPIQ score, range: 8-56, PSU cutoff = 40	26.52	(8.77)
	**%**	
Youth at cutoff for video game PSU	6.65	
Youth at cutoff for social media PSU	3.37	
Youth at cutoff for smartphone PSU	6.78	

Note: BAS = Behavioral Activation Scale; BIS = Behavioral Inhibition Scale; CBCL = Child Behavior Checklist; EF = executive functioning; GED = General Educational Development (certification); MPIQ = Mobile Phone Involvement Questionnaire; PSU = problematic screen use; SMAQ = Social Media Addiction Questionnaire; ST = screen time; VGAQ = Video Game Addiction Questionnaire.

### Measures

When youth were ages 9 to 10, baseline measures captured relevant characteristics before heavy screen use initiation. Impulsivity was assessed via the UPPS-P impulsivity scale, an abbreviated version of the original UPPS scale, a reliable and valid impulsivity measure.^23^ The UPPS-P contains 20 items with Likert scale responses ranging from 1 (“Not at all like me”) to 4 (“Very much like me”). Total UPPS scores quantify broad impulsivity, with 4 subfacet scales—urgency, sensation-seeking, lack of planning, and lack of perseverance—providing specificity. Although a 4-factor model fit the original UPPS scale best, a fifth subfacet—positive urgency—was later added and retained by the ABCD Study. Subsequent factor analyses suggested that positive and negative urgency represent 1 construct. Additionally, positive urgency has been associated with elevated negative affect and decreased positive affect and does not moderate associations between positive affect and impulsivity.^36^ Thus, urgency was treated as 1 factor.

RS and PS were operationalized through self-reported scores on a modified version of the Behavioral Inhibition Scale/Behavioral Activation Scale optimized for use in adolescents.^26^ Here, RS was operationalized as total scores on the Drive and Reward Responsiveness subscales combined. The Fun-Seeking scale was excluded because its items load poorly in adolescents and because removing Fun-Seeking items improves Behavioral Activation Scale factor fit.^26^ PS was operationalized as total Behavioral Inhibition Scale scores.

Total raw scores on the Internalizing and Externalizing Problems scales of the school-age, parent-reported Child Behavior Checklist (CBCL)^37^ were quantified and then log-transformed to reduce skew in the distributions. The Internalizing Problems scale sums scores from the Anxious/Depressed, Withdrawn/Depressed, and Somatic Complaints subscales. The Externalizing Problems scale sums scores from the Rule-Breaking and Aggressive Behavior subscales.

Neurocognitive abilities were operationalized by general cognitive ability, EF, and learning/memory components derived from a principal components analysis of 9 cognitive tasks (the Little Man Task, the Rey Auditory Verbal Learning Task, and 7 NIH Toolbox Cognitive battery tasks) administered to the ABCD sample at baseline.^35^ The NIH Toolbox Picture Vocabulary and Oral Reading tests loaded most strongly onto the first component (general cognitive ability); the NIH Toolbox Flanker, NIH Toolbox Pattern Comparison Processing Speed, and NIH Toolbox Dimensional Change Card Sort tasks loaded most strongly onto the EF component; and the NIH Toolbox Picture Sequence Memory task and Rey Auditory Verbal Learning Test loaded most strongly onto the learning/memory component.

ST was operationalized via self-report responses to the ABCD Screen Time Questionnaire (STQ)^38^ at the baseline and year 4 waves. Video game ST was operationalized as total average hours spent playing both single-player and multiplayer video games on a typical day, and social media ST was operationalized as total average hours spent on social media applications on a typical day. Total daily average values across weekdays and weekends were quantified, similar to other studies.^5^ Sensitivity analyses were conducted to assess differences between predictors of weekday vs weekend ST hours (Table S1, available online). Although smartphone/smartwatch ownership was queried, the ABCD Study did not measure self-reported hours of smartphone ST.

PSU measures were administered at year 4, but not at baseline. PSU was operationalized via the Video Game Addiction Questionnaire (VGAQ),^38^ Social Media Addiction Questionnaire (SMAQ),^38^ and Mobile Phone Involvement Questionnaire (MPIQ),^38^ which were based on the Bergen Facebook Addiction Scale, a valid, reliable questionnaire assessing 6 core addictive processes (salience, mood modification, tolerance, withdrawal, conflict, and relapse) of Facebook use.^7^ The VGAQ and SMAQ each have 6 items with Likert scale responses ranging from 1 (“Never”) to 6 (“Very often”). Each item assesses 1 of 6 symptoms of addiction. The MPIQ also measures 1 unitary construct and has 8 items reflecting these 6 symptoms, plus 2 items reflecting social motivations for phone usage and disruption of daily life owing to phone usage. MPIQ answer options are Likert scale responses ranging from 1 (“Strongly disagree”) to 7 (“Strongly agree”). PSU scores were continuous; for descriptive purposes (Table 1), participants were categorized as problematic or nonproblematic users of the 3 activities examined, with cutoffs based on previous work in adolescents.^18^

### Statistical Approach

Statistical analyses tested whether baseline individual differences in each of the 4 domains (impulsivity, motivation, psychopathology, cognition) at ages 9 to 10 predicted average daily hours of ST of video games and social media and PSU of video games, social media, and phones at ages 12 to 15. The impulsivity subfacets (urgency, sensation-seeking, lack of planning, and lack of perseverance) were entered as predictors in the same model, yielding estimates of each subfacet effect while controlling for the others. All other motivation, psychopathology, and cognition characteristics were modeled as independent predictors of screen use. All analyses implemented linear mixed effects models run using the R studio lme4 package.^39^

Analyses controlled for race/ethnicity, child sex, baseline age, baseline household income, and baseline maximum parental education, as these demographics and socioeconomic status variables are known to affect screen use in this age group and in the ABCD sample specifically.^5^ Because some participants were siblings, and because participants were assessed at 21 different sites, analyses modeled family and study site as nested random effects. To reduce false discoveries, Bonferroni corrections penalized *p* values based on the number of tests within each domain. For example, within cognition, the standard significance level of .05 was divided by the number of tests in the domain (3). CBCL internalizing and externalizing raw scores were log-transformed before being entered into models, as CBCL items had considerable skewness. Missing data were handled through listwise deletion. Missingness was low: there were no missing impulsivity values and very few missing motivation (0.15%) or psychopathology (0.02%) values. Approximately 7.8% of cognition values were missing.

## Results

### Descriptive Statistics

Demographic, screen use, and individual difference summary statistics are shown in Table 1. The sample is 52% male and 48% female, and the sample’s racial demographics closely approach parity with American youth census data at the time of data collection.^34^ At baseline, 99.77% of the sample were 9 to 10 years old; at year 4, 99.85% of the sample were 12 to 15 years old. The sample’s socioeconomic status was somewhat above average: 68.63% of youth had at least 1 parent with a bachelor’s degree or higher.

At ages 9 to 10, the sample was largely naïve to lengthy screen use (Table 1). At ages 12 to 15, screen use was considerably higher; 65% of the sample now played video games for more than 30 minutes daily, with average daily hours spent on video games increasing by nearly 2 hours. Whereas only 17% of the sample used social media at baseline, by year 4, 62% of the sample did, with typical daily social media hours now exceeding 90 minutes. By year 4, 89% of the sample reported smartphone/smartwatch ownership. However, despite increasing use, only a minority of participants in the sample (approximately 3%-7%, varying by activity) met cutoffs for PSU.

Table 2 presents zero-order correlations among measures. Notably, at year 4, hours spent playing video games and using social media correlated (*r* = 0.28 and *r* = 0.27, respectively) with concurrent VGAQ and SMAQ scores, supporting the idea that a high number of daily hours engaged in ST does not necessarily indicate PSU.

**Table 2:** Intercorrelations Among Individual Difference, Screen Time (ST), and Problematic Screen Use (PSU) Measures

	U	LOPlan	SS	LOPers	BAS D + RR	BIS	CBCL-I	CBCL-E	GCA	EF	L/M	VG	VGAQ	SM	SMAQ	MPIQ
**U**	1	—	—	—	—	—	—	—	—	—	—	—	—	—	—	—
**LOPlan**	0.21∗∗∗	1	—	—	—	—	—	—	—	—	—	—	—	—	—	—
**SS**	0.19∗∗∗	0.07∗∗∗	1	—	—	—	—	—	—	—	—	—	—	—	—	—
**LOPers**	0.19∗∗∗	0.45∗∗∗	−0.08∗∗∗	1	—	—	—	—	—	—	—	—	—	—	—	—
**BAS D + RR**	0.24∗∗∗	−0.01	0.18∗∗∗	−0.08∗∗∗	1	—	—	—	—	—	—	—	—	—	—	—
**BIS**	0.23∗∗∗	0.02	0.02	−0.05∗∗∗	0.27∗∗∗	1	—	—	—	—	—	—	—	—	—	—
**CBCL-I**	0.08∗∗∗	0.03	−0.05∗∗∗	0.10∗∗∗	0.00	0.09∗∗∗	1	—	—	—	—	—	—	—	—	—
**CBCL-E**	0.18∗∗∗	0.13∗∗∗	0.02	0.13∗∗∗	0.11∗∗∗	0.01	0.58∗∗∗	1	—	—	—	—	—	—	—	—
**GCA**	−0.14∗∗∗	0.05∗∗∗	0.11∗∗∗	−0.05∗∗∗	−0.14∗∗∗	−0.02	−0.01	−0.12∗∗∗	1	—	—	—	—	—	—	—
**EF**	−0.04∗∗∗	0.02	0.04∗	−0.08∗∗∗	−0.03∗	−0.02	−0.05∗∗∗	−0.07∗∗∗	0.17∗∗∗	1	—	—	—	—	—	—
**L/M**	−0.11∗∗∗	0.00	0.00	−0.09∗∗∗	−0.12∗∗∗	0.00	−0.03	−0.11∗∗∗	0.32∗∗∗	0.16∗∗∗	1	—	—	—	—	—
**VG**	0.15∗∗∗	0.04∗∗∗	0.02	0.10∗∗∗	0.13∗∗∗	0.00	0.04∗∗∗	0.12∗∗∗	−0.2∗∗∗	−0.11∗∗∗	−0.17∗∗∗	1	—	—	—	—
**VGAQ**	0.10∗∗∗	0.05∗∗∗	0.00	0.08∗∗∗	0.05∗∗∗	0.06∗∗∗	0.06∗∗∗	0.06∗∗∗	0.01	−0.02	−0.05∗∗∗	0.28∗∗∗	1	—	—	—
**SM**	0.09∗∗∗	0.01	−0.01	0.02	0.12∗∗∗	0.04∗	0.02	0.05∗∗∗	−0.18∗∗∗	−0.05∗∗∗	−0.11∗∗∗	0.27∗∗∗	0.00	1	—	—
**SMAQ**	0.05∗∗∗	0.02	−0.02	0.03	0.02	0.06∗∗∗	0.03∗	0.02	−0.02	−0.02	−0.02	0.04∗	0.24∗∗∗	0.27∗∗∗	1	—
**MPIQ**	0.11∗∗∗	0.04∗	0.01	0.06∗∗∗	0.07∗∗∗	0.09∗∗∗	0.03	0.04∗	−0.05∗∗∗	−0.02	−0.04∗	0.05∗∗∗	0.17∗∗∗	0.28∗∗∗	0.46∗∗∗	1

Note: All values are zero-order Pearson’s r correlations. Individual differences were measured at baseline; ST and PSU measures were assessed at year 4. BAS D + RR = Behavioral Activation Scale Drive + Reward Responsiveness; CBCL-E = Child Behavior Checklist Externalizing; CBCL-I = Child Behavior Checklist Internalizing; EF = executive functioning; GCA = general cognitive ability; L/M = learning/memory; LOPers = Lack of Perseverance; LOPlan = Lack of Planning; SM = social media hours; SMAQ = Social Media Addiction Questionnaire; SS = sensation-seeking; U = baseline urgency; VG = video game hours; VGAQ = Video Game Addiction Questionnaire.

∗ *p* < .05; ∗∗∗*p* < .001.

### Predictors of Daily Hours of Screen Time

Table 3 shows main effects from linear mixed effects models testing baseline individual difference predictors of later average daily hours of ST, controlling for relevant above-described covariates.

**Table 3:** Main Effects of Baseline Individual Difference Predictors of Later Screen Time (ST)

Domain	Video game hours				Social media hours			
Impulsivity (∝ = 0.05)	**Predictor**	**β**	**SE**	***p***	**Predictor**	**β**	**SE**	***p***
	**Urgency**	**+.07**	**0.01**	**<.001**	**Urgency**	**+.06**	**0.01**	**<.001**
	Sensation-seeking	−.00	0.02	.740	Sensation-seeking	+.01	0.01	.571
	Lack of planning	−.01	0.02	.601	Lack of planning	+.03	0.01	.061
	**Lack of perseverance**	**+.07**	**0.02**	**<.001**	Lack of perseverance	+.02	0.02	.290
Motivation (∝ = 0.025)^a^	**Reward sensitivity**	**+.04**	**0.01**	**<.001**	**Reward sensitivity**	**+.08**	**0.01**	**<.001**
	Punishment sensitivity	+.02	0.02	.094	Punishment sensitivity	+.02	0.01	.285
Psychopathology (∝ = 0.025)^a^	**Internalizing**	**+.03**	**0.01**	**.017**	Internalizing	+.01	0.01	.387
	**Externalizing**	**+.04**	**0.01**	**.002**	**Externalizing**	**+.04**	**0.01**	**.008**
Cognition (∝ = 0.017)^a^	**General cognitive ability**	**−.10**	**0.08**	**<.001**	**General cognitive ability**	**−.08**	**0.05**	**<.001**
	Executive functioning	−.03	0.07	.018	Executive functioning	−.03	0.04	.072
	**Learning/memory**	**−.05**	**0.07**	**<.001**	**Learning/memory**	**−.06**	**0.05**	**<.001**

Note: Boldface type indicates significant results after Bonferroni correction within domain.

a Bonferroni-corrected α threshold is listed per domain.

#### Video Game Use

Video game hours were predicted by individual differences within all 4 domains. Within the domain of impulsivity, video game hours were predicted by higher urgency and lack of perseverance, but not by sensation-seeking scores. Higher baseline RS was associated with higher year 4 daily hours of video game use. Both higher internalizing and higher externalizing were associated with higher later daily hours of video game use. Lower baseline EF did not predict later daily hours of video game use, though lower general cognitive ability and learning/memory scores did.

#### Social Media Use

As with video game ST, higher urgency scores, but not other impulsivity facets, predicted greater daily hours of social media use. Both higher baseline RS and externalizing were associated with more hours spent using social media at year 4. Lower baseline general cognitive ability and learning/memory scores, but not EF, were associated with more hours of social media use at year 4.

Sensitivity analyses were conducted to determine whether these findings for average daily ST varied by weekday vs weekend use. As indicated in Table S1, available online, patterns are largely unchanged.

### Predictors of Problematic Screen Use

Predictors of problematic video game use, social media use, and phone use are summarized in Table 4.

**Table 4:** Main Effects of Baseline Individual Difference Predictors of Later Problematic Screen Use

Domain	Problematic video game use				Problematic social media use				Problematic smartphone use			
Impulsivity (∝ = 0.05)	**Predictor**	**β**	**SE**	***p***	**Predictor**	**β**	**SE**	***p***	**Predictor**	**β**	**SE**	***p***
	**Urgency**	**+.08**	**0.02**	**<.001**	**Urgency**	**+.05**	**0.02**	**<.001**	**Urgency**	**+.10**	**0.01**	**<.001**
	**Sensation-seeking**	**−.04**	**0.03**	**.015**	Sensation-seeking	−.01	0.03	.730	Sensation-seeking	+.02	0.01	.172
	Lack of planning	+.00	0.04	.872	Lack of planning	+.02	0.04	.195	Lack of planning	+.02	0.01	.122
	**Lack of perseverance**	**+.04**	**0.05**	**.029**	Lack of perseverance	+.02	0.04	.258	Lack of perseverance	+.05	0.02	.003
Motivation (∝ = 0.025)^a^	Reward sensitivity	+.01	0.02	.372	Reward sensitivity	+.03	0.02	.038	**Reward sensitivity**	**+.08**	**0.01**	**<.001**
	**Punishment sensitivity**	**+.07**	**0.03**	**<.001**	**Punishment sensitivity**	**+.04**	**0.03**	**.002**	**Punishment sensitivity**	**+.06**	**0.01**	**<.001**
Psychopathology (∝ = 0.025)^a^	**Internalizing**	**+.06**	**0.01**	**.001**	Internalizing	+.01	0.01	.332	Internalizing	+.02	0.01	.230
	**Externalizing**	**+.04**	**0.01**	**.007**	Externalizing	+.03	0.01	.040	**Externalizing**	**+.05**	**0.01**	**<.001**
Cognition (∝ = 0.017)^a^	General cognitive ability	+.04	0.15	.025	General cognitive ability	+.01	0.13	.650	General cognitive ability	+.00	0.05	.869
	Executive functioning	−.00	0.13	.858	Executive functioning	−.03	0.12	.089	Executive functioning	−.02	0.04	.162
	Learning/memory	−.01	0.14	.651	Learning/memory	−.02	0.13	.234	Learning/memory	−.03	0.05	.086

Note: Boldface indicates significant results after Bonferroni correction within domain.

a Bonferroni-corrected α threshold is listed per domain.

#### Problematic Video Game Use

Problematic video game use (VGAQ score) was predicted by higher urgency and lack of perseverance scores. Counterintuitively, higher sensation-seeking scores predicted lower year 4 VGAQ scores. High baseline PS predicted higher year 4 VGAQ scores. RS was not significantly related to year 4 VGAQ scores. As with video game ST, both internalizing and externalizing scores at baseline predicted higher year 4 VGAQ scores. No cognition variables predicted year 4 VGAQ scores.

#### Problematic Social Media Use

Higher baseline urgency and PS scores predicted greater SMAQ scores. Neither baseline psychopathology nor aspects of cognition predicted later SMAQ scores.

#### Problematic Phone Use

Problematic phone use (MPIQ) scores were predicted by higher baseline urgency and lack of perseverance scores. MPIQ scores were the only PSU scores predicted by both high RS and high PS as well as by high externalizing, but not internalizing. Baseline cognition was unrelated to later MPIQ scores.

## Discussion

This study assessed whether risk factors that have been associated with SUDs^10^ predict PSU in a large adolescent sample, focusing on impulsivity, motivation, psychopathology, and cognition. Youth with higher urgency, higher RS, higher externalizing, and lower cognitive abilities in late childhood reported a greater number of daily average ST hours in mid-adolescence. However, a greater number of hours of daily engagement with screen activities did not necessarily imply PSU, given that the 2 variables were only modestly correlated. Rather, across screen activities, PSU was consistently predicted by higher urgency and PS, but not by characteristics related to disinhibition and RS. Some PSU results varied by activity.

Cross-sectional studies find individuals with PSU to be more impulsive.^15^, ^16^, ^17^ The current findings suggest that relatively high levels of urgency, but not other forms of impulsivity, may predispose some youth toward developing increasingly compulsive screen use. Prospective associations of urgency with all forms of later ST and PSU examined suggest that it is capturing a nonspecific vulnerability factor for screen use. In the context of high positive or negative emotion, it may be that screen activities satisfy urges for distraction and escapism as youth attempt to self-regulate. In other contexts, youth with high levels of urgency prospectively report increased emotional dysregulation.^40^ These youth may be particularly vulnerable to adverse long-range effects of screen use. Although urgency was associated with both average daily hours of ST and PSU in the current study, PS predicted PSU alone, suggesting that motivation and reinforcement patterns could be mechanisms connecting these traits to PSU risk. One meta-analysis of 115 studies and 40,432 participants assessed with the UPPS found that urgency was strongly correlated with psychopathology, with the strongest effects for depression and anxiety.^24^ Youth with high PS and high urgency may experience more frequent episodes of anxiety than youth with high urgency alone. Screen activities may provide quick and easy relief from anxiety,^41^ yielding temporary emotional distraction that becomes negatively reinforcing in the long term. Over time, it is possible that this reinforcement pattern may become a vicious cycle, leading youth to rely on screen activities to self-soothe in the face of distress. Although trait interactions were not assessed here, previous research has found emotion-focused coping to mediate positive associations between high PS and higher problematic internet use scores,^42^ supporting the speculation that urgency and PS combined could create problematic motivations for screen use. In addition, despite the possibility that these youth engage in excessive screen use as a form of avoidance, it is also possible that screen use may be acutely associated with increases, rather than decreases, in anxiety symptoms. High levels of screen use have been prospectively associated with greater levels of internalizing^43^, ^44^, ^45^ as well as comorbid internalizing/externalizing psychopathology in youth.^44^ Thus, urgency and PS may reflect vulnerability to later PSU and subsequent internalizing psychopathology in this young sample. Taking real-time measurements of motivations for screen use using ecological momentary assessments could test this theory, assessing whether screen-based coping behaviors follow a pattern of temporary negative reinforcement or whether they are, in fact, anxiety-inducing. When studying long-range consequences of screen use, researchers will ideally account for preexisting differences in urgency.

Importantly, the finding of the current study suggest that developmental pathways to PSU may differ markedly from developmental pathways to SUDs. Although impulsivity is recognized as an SUD risk factor,^10^ its association with PSU was specific to certain subfacets, and some of these patterns were unexpected. For instance, whereas sensation-seeking is distinctly associated with SUDs,^24^ sensation-seeking either was unrelated to PSU or was even associated with less PSU (as with video games). Although video gaming may allow simulation of sensation-seeking behaviors (eg, using firearms and driving recklessly), rewards and punishments in video games are only symbolic, perhaps preventing screen activities from feeling truly thrilling or strongly physiologically arousing. This interpretation is speculative, given that the current study did not directly query youth about their arousal levels in the context of active screen usage. It is also possible that the sensation-seeking items on the modified UPPS-P fail to capture aspects of sensation-seeking that are most relevant to problematic screen use.^46^

Additionally, though externalizing is an established risk factor for SUDs of various substances,^47^ externalizing predicted some, but not all, forms of PSU. Likewise, whereas low EF predicts substance use,^32^ EF skills at ages 9 to 10 were unrelated to all forms of screen use assessed at ages 12 to 15. These are important observations that challenge the notion that addictions share a common etiology.^10^^,^^20^

These patterns also raise questions about how PSU might be best categorized in overarching structures of psychopathology, such as the Hierarchical Taxonomy of Psychopathology (HiTOP), a dimensional and empirically validated system.^47^ SUD is placed under the HiTOP disinhibited externalizing spectrum. The current study, however, links PSU to both externalizing- and internalizing-related characteristics.

Interestingly, some variation by specific screen activity was observed. Problematic video game use was the only form of PSU predicted by internalizing, and problematic phone use was uniquely predicted by externalizing. Unique internalizing associations with video game PSU may exist owing to opportunities for immersive solitary play on video game platforms, whereas externalizing links with phone PSU may exist owing to opportunities for socialization on smartphones. Thus, it is speculated that highly withdrawn children use video games to escape external stressors, whereas highly disinhibited children use smartphone applications to seek social stimulation.

The lack of effects observed between baseline internalizing and later social media use is surprising given that concurrent associations between internalizing and social media use have been described.^44^ It may be that the parent-reported CBCL failed to fully capture the range of internalizing psychopathology in the baseline sample, particularly given the associations that were observed for self-reported PS and urgency. Alternatively, these patterns could support the idea that internalizing is a consequence, and not a predictor, of social media use, as suggested by others.^48^

This study has several strengths. The longitudinal sample allowed for testing of prospective associations between baseline individual differences and later screen use, rather than cross-sectional associations comparing characteristics of heavy and light screen users.^15^, ^16^, ^17^^,^^30^ The nuanced impulsivity measure of this study enabled testing of which specific impulsive traits contribute most to later PSU, revealing unique associations with urgency. Moreover, the specificity of associations of individual differences with 3 screen activities popular among teenagers was tested, assessing shared and specific predictors of PSU across media types. Finally, the individual difference measures of the ABCD Study are highly comprehensive, with self-report, parent-report, and behavioral measures assessing personality, psychopathology, and cognition.

Nonetheless, limitations of this study require acknowledgment. Personality, motivation, and screen use measures were self-reported, making them vulnerable to recall bias. Self-reported ST measures may have also lacked precision, considering that children may lack awareness of their own ST. In 1 pilot study of passive-sensed smartphone ST in 67 ABCD participants, objective measurements of smartphone ST in children correlated (*r* = 0.49) only with self-reported smartphone ST; 79% of children underreported their ST.^49^ Parent-reported psychopathology measures may have also lacked precision, as significant discrepancies are evident between parent- and child-reported internalizing symptoms.^50^ Additionally, this analysis did not consider potential trait interactions or sex differences in developmental pathways to PSU. Moreover, this analysis relied on 2 ABCD assessment waves. Notably, ABCD waves 2 and 3 coincided with the COVID-19 pandemic, which differentially impacted youth at various sites based on geopolitical factors. Prior analyses of the wave 2 sample found that 7% of screen users ages 11 to 12 reported video game PSU, 3.5% reported social media PSU, and 21.8% reported smartphone PSU—rates that are highly similar to what was observed here at year 4, with the exception of smartphone PSU.^18^ Analyses that focus specifically on impacts of the COVID-19 pandemic on screen use patterns and later screen use outcomes would be instructive, but are beyond the scope of this report.

Limitations notwithstanding, the current findings have important real-world implications for parents, clinicians, and policymakers. Parents involved in monitoring their children’s screen use may wish to focus their energies on children with emotional dysregulation or anxious personalities. Given the possibility that links of urgency with PSU may be explained by children using screens to cope with strong emotions, parents might encourage their children to adopt coping strategies that do not involve screens. Similarly, clinicians treating young people may consider incorporating assessment of daily screen time hours and PSU symptoms alongside routine assessments of internalizing symptoms. Clinicians may be able to detect emerging PSU behaviors and provide personalized interventions early in adolescence, incorporating digital media behaviors into intervention planning. Policymakers considering regulation of digital media platforms targeting young audiences could consider that limitations on screen use in early to mid-adolescence may not be universally necessary but may instead be targeted toward vulnerable youth. For instance, built-in protections on digital media platforms, such as pop-up banners directing younger users toward mental health resources after long periods of time on a website, may be impactful.

Engagement with digital media for long hours each day does not necessarily imply that an adolescent’s screen use is problematic. Rather, youth with relatively high levels of traits related to anxious distress appear more likely to develop PSU. Distress-prone youth and youth who tend to respond to this distress more rashly or have general difficulties with emotion regulation may benefit from parental monitoring and guidance of their screen use. Additional research should be conducted to explore the differences in PSU from other addictive behaviors given that a distinct pattern of premorbid risk is evident.

## CRediT authorship contribution statement

**Beatrice A. Grund:** Writing – review & editing, Writing – original draft, Visualization, Methodology, Investigation, Formal analysis, Data curation, Conceptualization. **Monica Luciana:** Writing – review & editing, Methodology, Conceptualization.
