# Supplementary material for: Prospective Predictors of Adolescent Screen Time and Problematic Screen Use
Source: JAACAP Open. 2025 Jul 3;3(4):1259–69. doi: 10.1016/j.jaacop.2025.06.007 (PMC12684653; doi:10.1016/j.jaacop.2025.06.007)
Supplement: Supplemental Table S1 [file mmc1.docx]

**Supplemental Table 1**

*Main effects of baseline individual difference predictors on year 4 weekday and weekend screen time*

| **Domain** | **Video game hours: Weekday** | | | | **Video game hours: Weekend** | | | |
| --- | --- | --- | --- | --- | --- | --- | --- | --- |
| Impulsivity  ($\propto$ = 0.05) | *Predictor* | *S Beta* | *SE* | *p* | *Predictor* | *S Beta* | *SE* | *p* |
|  | Urgency | +0.09 | 0.01 | <0.001 | Urgency | +0.07 | 0.01 | <0.001 |
|  | Sensation-  Seeking | -0.00 | 0.01 | 0.677 | Sensation-  Seeking | -0.00 | 0.01 | 0.955 |
|  | Lack of  Planning | +0.01 | 0.01 | 0.120 | Lack of  Planning | +0.02 | 0.01 | 0.284 |
|  | Lack of  Perseverance | +0.07 | 0.01 | <0.001 | Lack of  Perseverance | +0.08 | 0.01 | <0.001 |
| Motivation  ($\propto$ = 0.025) | Reward  Sensitivity | +0.04 | 0.01 | 0.002 | Reward  Sensitivity | +0.04 | 0.01 | 0.002 |
|  | Punishment  Sensitivity | +0.02 | 0.01 | 0.092 | Punishment  Sensitivity | +0.02 | 0.01 | 0.170 |
| Psychopathology  ($\propto$ = 0.025) | Internalizing | +0.02 | 0.01 | 0.043 | Internalizing | +0.03 | 0.01 | 0.009 |
|  | Externalizing | +0.03 | 0.01 | 0.136 | Externalizing | +0.05 | 0.01 | <0.001 |
| Cognition  ($\propto$ = 0.017) | General  Cognitive Ability | -0.10 | 0.02 | <0.001 | General  Cognitive Ability | -0.05 | 0.02 | 0.002 |
|  | Executive  Functioning | -0.03 | 0.01 | 0.020 | Executive  Functioning | -0.03 | 0.01 | 0.039 |
|  | Learning/  Memory | -0.06 | 0.01 | <0.001 | Learning/  Memory | -0.02 | 0.01 | 0.125 |

| **Domain** | **Social Media hours: Weekday** | | | | **Social Media hours: Weekend** | | | |
| --- | --- | --- | --- | --- | --- | --- | --- | --- |
| Impulsivity  ($\propto$ = 0.05) | *Predictor* | *S Beta* | *SE* | *p* | *Predictor* | *S Beta* | *SE* | *p* |
|  | Urgency | +0.07 | 0.01 | <0.001 | Urgency | +0.05 | 0.01 | <0.001 |
|  | Sensation-  Seeking | +0.02 | 0.01 | 0.185 | Sensation-  Seeking | +0.02 | 0.01 | 0.139 |
|  | Lack of  Planning | +0.02 | 0.01 | 0.549 | Lack of  Planning | -0.01 | 0.01 | 0.615 |
|  | Lack of  Perseverance | +0.04 | 0.01 | 0.001 | Lack of  Perseverance | +0.03 | 0.01 | 0.063 |
| Motivation  ($\propto$ = 0.025) | Reward  Sensitivity | +0.08 | 0.01 | <0.001 | Reward  Sensitivity | +0.08 | 0.01 | <0.001 |
|  | Punishment  Sensitivity | +0.02 | 0.01 | 0.256 | Punishment  Sensitivity | +0.01 | 0.01 | 0.406 |
| Psychopathology  ($\propto$ = 0.025) | Internalizing | +0.01 | 0.01 | 0.383 | Internalizing | +0.01 | 0.01 | 0.452 |
|  | Externalizing | +0.04 | 0.01 | 0.007 | Externalizing | +0.03 | 0.01 | 0.027 |
| Cognition  ($\propto$ = 0.017) | General  Cognitive Ability | -0.08 | 0.01 | <0.001 | General  Cognitive Ability | -0.06 | 0.02 | <0.001 |
|  | Executive  Functioning | -0.05 | 0.02 | 0.064 | Executive  Functioning | -0.05 | 0.02 | 0.120 |
|  | Learning/  Memory | -0.07 | 0.02 | <0.001 | Learning/  Memory | -0.04 | 0.01 | 0.014 |

*Note*. * = Bonferroni-corrected alpha listed. Significant results highlighted in light grey. S Beta = standardized beta, SE = standard error.
